# Nasal Involvement in Obstructive Sleep Apnea Syndrome

**DOI:** 10.1155/2014/717419

**Published:** 2014-11-20

**Authors:** Daniel de Sousa Michels, Amanda da Mota Silveira Rodrigues, Márcio Nakanishi, André Luiz Lopes Sampaio, Alessandra Ramos Venosa

**Affiliations:** ^1^Department of Otorhinolaryngology and Head and Neck Surgery, Brasília University Hospital, HUB, SGAN 605, Avenida L2 Norte, 70830-200 Brasília, DF, Brazil; ^2^Universidade de Brasília (UnB), Campus Universitário Darcy Ribeiro, 70910-900 Brasília, DF, Brazil

## Abstract

Numerous studies have reported an association between nasal obstruction and obstructive sleep apnea syndrome (OSAS), but the precise nature of this relationship remains to be clarified. This paper aimed to summarize data and theories on the role of the nose in the pathophysiology of sleep apnea as well as to discuss the benefits of surgical and medical nasal treatments. A number of pathophysiological mechanisms can potentially explain the role of nasal pathology in OSAS. These include the Starling resistor model, the unstable oral airway, the nasal ventilatory reflex, and the role of nitric oxide (NO). Pharmacological treatment presents some beneficial effects on the frequency of respiratory events and sleep architecture. Nonetheless, objective data assessing snoring and daytime sleepiness are still necessary. Nasal surgery can improve the quality of life and snoring in a select group of patients with mild OSAS and septal deviation but is not an effective treatment for OSA as such. Despite the conflicting results in the literature, it is important that patients who are not perfectly adapted to CPAP are evaluated in detail, in order to identify whether there are obstructive factors that could be surgically corrected.

## 1. Introduction

In “Morbis Popularibis,” Hippocrates observed that nasal polyps were associated with restless sleep [1]. In practice, most people experience difficulty sleeping during episodes of nasal congestion associated with upper airways infections. Thus, a link between nasal breathing and sleep, as well as an improvement in sleep quality after a relief of the nasal obstruction, appears to be intuitive [2].

Many nose and pharynx abnormalities may cause or worsen snoring and sleep apnea, such as septal deviation, nasal polyps, turbinate hypertrophy, and rhinitis. Adenoid hypertrophy, nasopharyngitis, and nasopharyngeal tumors can also cause narrowing of the airways in the nasopharyngeal region [3].

Epidemiological studies have demonstrated a relationship between the measure of nasal airflow and snoring [4], but attempts to find a linear correlation between nasal obstruction and sleep apnea have been less successful [5]. A weak correlation between nasal resistance measured by posterior rhinomanometry and severity of sleep apnea has been recently reported [6].

This paper aimed to summarize data and theories on the role of the nose in the pathophysiology of sleep apnea as well as to discuss the benefits of surgical and medical nasal treatments.

## 2. Nasal Involvement in the Pathophysiology of Obstructive Sleep Apnea Syndrome (OSAS)

The nose accounts for over 50% of the total upper airway resistance and plays an important role in the establishment of physiological functions such as humidification, heating, and air filtration [7].

Among the places of greatest resistance to nasal airflow are the vestibule and nasal valve area, determined by the alar cartilages, septum, and inferior turbinates [8].

The nasal mucosa is a dynamic organ controlled by the autonomic nervous system. Periodic nasal congestion and decongestion have been termed the “nasal cycle” by Heetderks [9]. This cycle occurs in approximately 80% of the adult population. In patients with permanent unilateral nasal obstruction, the nasal cycle may contribute to a significant increase in total airway resistance [10].

In healthy individuals, the lateral decubitus increases congestion in the ipsilateral nasal cavity and reduces airflow resistance in the contralateral nasal cavity. This does not occur due to a hydrostatic effect, but rather as a reflex response caused by asymmetric pressure on the body [11, 12]. This reflex interrupts the nasal cycle [13]. However, in the assessment of both nasal cavities, no significant changes were observed in the cross-sectional area when comparing supine and lateral decubitus [14].

To fully explain the relationship between airflow, nasal obstruction, and sleep apnea, it is necessary to understand some dynamic theories of physics.

Among the physiological mechanisms that elucidate the relationship between nasal airflow and breathing during sleep are the Starling resistor model, the unstable oral airway proposition, the nasal respiratory reflex, and the role of nitric oxide (NO) [2].

**Figure 1:**
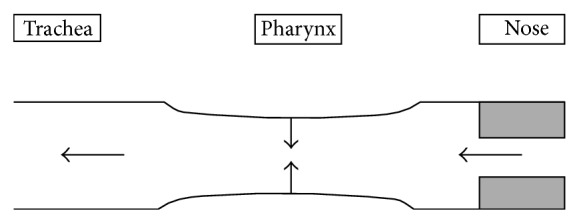
Starling resistor model.

According to the Starling resistor model (Figure 1), the upper airways function as a hollow tube with a constriction near the entrance hole, which corresponds to the nostrils, and a posterior collapsible segment, which corresponds to the oropharynx. This model predicts that the presence of a further upstream obstructive factor (nose) will generate a suction force, that is, a negative intraluminal pressure downstream (oropharynx), resulting in pharyngeal collapse in predisposed individuals [15, 16].

A closed jaw and proper dental occlusion stabilize the flow in the upper airways [17]. When nasal resistance exceeds a certain level, an air bypass occurs and leads to mouth breathing, resulting in a decrease in the retroglossal dimension, due to the subsequent retraction of the tongue, narrowing of the pharyngeal lumen, and increased oscillation and vibration of the soft palate and redundant tissue of the pharynx [14]. This shift from nasal to oral breathing is physiologically disadvantageous to the individual, leading to an unstable breathing pattern [2].

Central respiratory events have also been increasingly associated with oral breathing during sleep. Tanaka and Honda [18] proposed that, after a switch to oral breathing during sleep, there is greater CO_2_ elimination during expiration, caused by an increase in respiratory stimulus. The increase in central apneas suggests that the nose plays an important role in the regulation of respiration and not only in the maintenance of airway patency.

A third factor is the nasal ventilatory reflex. An experimental application of local anesthetics in the nasal mucosa of healthy patients led to a significant increase of obstructive and central apnea episodes, of the same magnitude as those reported with complete nasal obstruction [19]. Similar results from other experiments [20] confirmed that the activation of nasal receptors during nasal breathing had a direct positive effect on spontaneous ventilation, leading to a higher resting breathing frequency and minute ventilation. Mouth breathing reduces the activation of these nasal receptors, leading to deactivation of the nasal-respiratory reflex and reduction of spontaneous ventilation, which can trigger respiratory events in susceptible individuals with subclinical OSAS [21] or exacerbate apnea episodes [22].

Finally, NO appears to play a role in maintaining the patency of the upper airways, as a transmitter between the nose, pharyngeal muscles, and lungs [23]. NO is produced in significant quantities in the nose and in the paranasal sinuses and has been proven (including in clinical practice) to be a potent pulmonary vasodilator, improving oxygenation and ventilation-perfusion ratio [24]. As the total amount of inspired NO varies according to the nasal flow [25], it appears logical that a decrease in nasal breathing would result in reduction of NO delivery to the lungs and a reduction in blood oxygenation. NO also plays a role in the maintenance of muscle tone, regulation of neuromuscular pathways in the pharyngeal muscles, spontaneous respiration, and sleep regulation. In general, the role of NO in the regulation of nasal OSAS, although probably significant, is still not completely understood [26].

The nasal pathophysiology in the pathogenesis of OSAS is summarized in Table 1.

## 3. Clinical and Experimental Evidence

Evidence suggests that experimental reduction of nasal patency and flow has a significant effect on breathing during sleep.

Suratt et al. [27] induced obstructive apneas in healthy subjects through nasal occlusion with gauze. Lavie et al. [28] investigated the influence of partial and complete obstruction of the nose in healthy subjects and observed a significant increase in the number of apneas during sleep.

Evidence from several observational and cross-sectional studies demonstrates that the objective increase in measures of nasal resistance and the presence of allergic rhinitis (AR) are associated with OSAS. Lofaso et al. [6] performed posterior rhinomanometry in 528 patients and observed an increased nasal resistance in OSAS patients compared to the control group. In a large population study, Young et al. [29] identified chronic nasal congestion as a risk factor for OSAS.

Data from an electromyographic study in healthy men performed by Basner et al. [30] demonstrated that the upper airway tone is lower during oral breathing than during nasal breathing, suggesting that the activity of the dilator muscles of the upper airway can be modulated by receptors in the nasal mucosa, sensitive to airflow or pressure. However, two other studies demonstrated that the airway did not affect the electromyographic activity of the genioglossus muscle in normal individuals, but other pharyngeal dilator muscles have not been studied [31, 32].

Epidemiological studies [33] demonstrated that AR affects 9% to 42% of the population. The mechanism through which allergic rhinitis causes poor quality of sleep and daytime fatigue is not entirely clear, but it is believed that several factors are involved. Inflammatory mediators such as interferon- (IFN-) gamma, tumor necrosis factor- (TNF-) alpha, interleukin- (IL-) 1b, IL-4, IL-10 [34], postural changes, and certain therapeutic agents, such as antihistamines, may have a direct impact on sleep regulation. One study described a direct association between nasal resistance and the severity of OSAS in patients with AR [35], as well as between nasal obstruction and subjective sleep quality and daytime sleepiness [36]. A recent study observed that both the AR and nonallergic rhinitis (NAR) are associated with impaired sleep quality and demonstrated complaints in up to 83% of patients with NAR [37].

In a recent review, McNicholas [38] attempted to synthesize these apparently conflicting results, indicating that reversible nasal obstruction is perhaps more closely associated with OSAS than with permanent nasal obstruction. This finding was observed in studies of patients with temporary obstruction (including AR and iatrogenic causes), which demonstrated a more consistent association with OSAS than studies of patients with structural abnormalities, such as a deviated nasal septum.

Thus, it appears that nasal obstruction, especially the reversible type (whether artificial or disease-induced), is associated with snoring and mild OSAS. Nonetheless, a direct correlation between the degree of nasal obstruction and the severity of OSAS has not been observed; nasal obstruction does not appear to be the main contributing factor in the majority of patients with moderate to severe OSAS.

These are some lines of evidence that, even with limited methodology, show how the nasal respiratory dysfunction influences sleep apnea.

## 4. Nasal Treatment in OSAS

Treatment options for cases with nasal obstruction include nasal dilators, medical treatment, and surgical intervention. Some clinical trials have been performed, aiming to analyze the therapeutic possibilities; however, the evaluation of the results was not uniformly objective. Many of the studies are not randomized, do not include a control group, and have a small sample size and relatively short follow-up period [39].

## 5. Conservative Treatment

In patients with nasal obstruction secondary to chronic rhinitis, the main cause of increased nasal resistance is edema and turbinate hypertrophy. Among the options for drug treatment are topical corticosteroids and sympathomimetic decongestants. These medications reduce the levels of inflammatory mediators, or even directly cause vasoconstriction, thereby leading to a decrease in nasal resistance and improved sleep [39].

There is only one randomized controlled trial assessing the effect of topical nasal corticosteroids in adult OSAS (Table 1) [40]. In that study, Kiely et al. evaluated the effect of topical nasal fluticasone for four weeks in 23 patients with chronic rhinitis: 13 had moderate to severe OSAS (mean apnea-hypopnea index [AHI]: 26.5) and ten patients were snorers without OSAS (mean AHI: 3). A reduction in AHI (mean: −6.5) and in nasal resistance was observed in the OSAS group after treatment with fluticasone when compared to placebo. However, there was no improvement in subjective sleep quality (reported by the patient, or improvement in the snoring intensity reported by the partner), in sleep architecture (no change in the duration of REM sleep), or in oxyhemoglobin desaturation index (desaturation between average and minimum levels) in either group.

These findings suggest that nasal obstruction due to allergic rhinitis favors worsening of sleep apnea and that treatment with topical corticosteroids can be somewhat beneficial in cases of mild to moderate OSAS.

Studies on nasal decongestants, such as oxymetazoline [28, 41], were limited and were inconclusive (Table 2). The results demonstrate that nasal decongestants, whether or not associated with nasal dilators, are not effective in the management of OSA, as no improvement was observed in the degree of daytime sleepiness or AHI [42–45]. As their clinical use is limited to only a few days, nasal decongestants cannot be used for OSAS management.

It can be concluded that pharmacological improvement in nasal patency in patients with OSAS and chronic nasal obstruction presents some beneficial effects on the frequency of respiratory events and sleep architecture. Nonetheless, objective data assessing snoring and daytime sleepiness are still necessary.

The use of nasal dilators is an option for increasing nasal patency in the narrowest part of the airway, the nasal valve. Two of the nasal dilators available in the market are an external device, Breathe Right (CNS Inc.; Bloomington, MN, USA), and an internal device, Nozovent (Prevancure AB; Frölunda, Sweden). Five studies with small sample were retrieved, three using Breathe Right and two using Nozovent (Table 3) [46–48, 50].

Nasal dilators are generally not recommended in patients with OSAS, but they may be beneficial for those with simple snoring associated with rhinitis and/or nasal valve stenosis. Since they are affordable devices with few side effects, they can be useful in some selected cases, especially as a conservative option for patients with indication for surgical correction of the nasal valve.

## 6. Surgical Treatment

Nasal obstruction in patients with OSAS may be caused by septal deviation, nasal polyps, and turbinate hypertrophy, among several other abnormalities. In this context, surgical interventions, such as septoplasty, rhinoseptoplasty, functional endoscopic sinus surgery, turbinectomy, and nasal valve surgery, appear to be a good therapeutic option [51].

There is also a group of patients who may benefit from surgical intervention not for curative purposes, but as an adjuvant treatment to improve the effectiveness of the main therapeutic option, continuous positive airway pressure (CPAP).

Li et al. [52, 53] recently addressed the role of nasal surgery in patients with snoring and OSAS from two different perspectives. Initially [52], the efficacy of nasal surgery for relief of snoring and OSAS in patients with septal deviation was assessed. The authors concluded that complete relief of snoring was achieved in only 12% of patients. Secondly, the improvement in quality of life after nasal surgery alone in patients with OSAS and nasal obstruction was assessed [53]. This parameter was evaluated through generic and disease-specific questionnaires. Li et al. concluded that it is possible to significantly improve the quality of life by correcting an obstructed nasal airway and thus substantiated the role of nasal surgery in treating these patients. Notwithstanding, despite the significant improvement observed in the quality of life parameters, there was no statistically significant improvement in the objective polysomnographic data. This discrepancy between objective and subjective outcomes was also observed in several other similar studies after nasal surgery alone for OSAS treatment. Verse et al. [54] studied a cohort of 26 patients, 19 of whom had OSAS and seven of whom were simple snorers. A variety of nasal surgical procedures, including rhinoplasty, septoplasty, endoscopic sinus surgery, and nasal valve surgery, were performed. They concluded that although nasal surgery significantly improved subjective sleep quality and daytime sleepiness, the surgical response rate in the apnea group was only 15%, based on objective parameters (AHI). Four patients presented worsening of sleep apnea, despite the reduction in arousal index. This paradoxical effect can be explained by the “first night effect,” which occurs when the patient has the initial, preoperative, sleep study for the first time, he does not sleep well and as a result the study it may not reflect the true severity of the sleep apnea. In the postoperative evaluation, when the patient has already adapted to the methodology of the examination, the severity of the problem becomes more evident.

Morinaga et al. [55] evaluated how the pharyngeal morphology affects the outcomes of nasal surgery in patients with OSAS and nasal obstruction. The morphological characteristics analyzed included degree of tonsillar hypertrophy, Mallampati grade, and retroglossal space. They concluded that the most favorable surgical outcomes were observed in subjects whose soft palate was positioned higher and/or had larger retroglossal dimensions. Conversely, Li et al. [52] found a relationship between the degree of tonsillar hypertrophy and surgical outcomes. The most important (and only) randomized study was conducted in 2007, by Koutsourelakis et al., in Athens [56], who divided 49 patients with septal deviation and OSAS into two groups; one group underwent septoplasty and the other, a sham surgery. Despite the subjective improvement in nasal patency, no objective changes were observed on AHI or daytime sleepiness, assessed by the Epworth sleepiness scale. However, in the septoplasty group, four patients (14.8%) responded to surgery, according to the Sher criteria (AHI reduction by 50% or more), and only one patient was disease-free (AHI < 5). It was concluded that nasal surgery can improve the quality of life and snoring in a select group of patients with mild OSAS and septal deviation. Nasal surgery is certainly not the most effective treatment for all patients with OSAS, but further studies can better define subgroups of patients who can benefit from the surgical procedure.

## 7. Nasal Obstruction and CPAP

CPAP is the treatment of choice for moderate or severe OSAS; however, the rate of adherence to this form of therapy is less than 70% [57]. Over 50% of CPAP users complain of significant nasal symptoms, such as nasal congestion, rhinorrhea, nasal dryness, and sneezing [58], which may become more significant if the patient presents any structural abnormality of the nose.

Since the CPAP mask is in contact with the nose, it is reasonable to assume that nasal alterations constitute a limitation to its use. The presence of functional or anatomical abnormalities in the nasal cavity may require greater CPAP pressure titration for the elimination of respiratory events, causing patient discomfort and hindering adaptation to the device. Nonetheless, attributing the low adherence to CPAP to a condition of increased nasal resistance is a controversial hypothesis.

Tárrega et al. [59] evaluated, through rhinomanometry, 125 patients with indication for CPAP therapy and observed no correlation between nasal resistance and CPAP adherence. Haddad et al. [60] investigated the contribution of nasal factors on CPAP adherence and observed that the highest values of body mass index (BMI), neck circumference, and AHI were found in the group of patients with good CPAP adherence, while nasal parameters such as rhinoscopy, nasofibroscopy, and acoustic rhinometry showed no differences between the groups with good or poor adherence.

Conversely, Morris et al. [61] observed a greater minimum cross-sectional area in the interior turbinate of patients with good CPAP adherence. These results were corroborated by Sugiura et al. [62], who observed that nasal resistance was lower in patients who used CPAP during titration polysomnography. These data demonstrate the need of good nasal patency during the patient's initial adaptation to the device. So et al. [63] also investigated 36 patients using acoustic rhinometry and observed that the sum of the nasal area was greater in the group of patients with good CPAP adherence, but only in those with AHI < 60/hour.

Despite the conflicting results in the literature, it is important that patients who are not perfectly adapted to CPAP are evaluated in detail, in order to identify whether there are obstructive factors that could be surgically corrected. In a group of patients who underwent radiofrequency turbinectomy, Powell et al. [64] demonstrated a subjective improvement of nasal obstruction, which, in turn, increased CPAP adherence. Similarly, Friedman et al. [65] showed a significant decrease in the levels of CPAP titration after nasal surgery alone. In that study, a reduction in pressure required for the cessation of obstructive events was observed in patients with mild, moderate, and severe OSAS. The average reduction in CPAP titration pressure was 9.3 cm H_2_O preoperatively to 6.7 cm H_2_O postoperatively. Nakata et al. [66] assessed 12 patients with nasal obstruction and severe OSAS who did not respond to treatment with CPAP. After nasal surgery, a significant decrease in nasal resistance and a significant increase in minimum O_2_ saturation during sleep were observed. However, no change was evidenced in AHI, despite the decrease in CPAP titration pressure from 16.8 cm H_2_O to 12 cm H_2_O in five patients.

## 8. Final Considerations

Despite the recent progress in the study of the relation between OSAS and nasal obstruction, there are still areas of doubt. Many of the studies investigating this relationship had an inappropriate sample size, poorly defined patient populations, inadequate control groups, and inappropriate techniques to objectively evaluate nasal resistance.

It is established that an improvement in nasal resistance, whether through surgery, medication, or use of nasal dilators, may improve self-reported sleep quality. However, these results are not always followed by improvement in polysomnographic parameters. Despite the lack of evidence demonstrating the success of nasal surgery as an isolated treatment for moderate and severe OSAS, surgical procedures that improve nasal patency have a role in relieving symptoms of simple snoring and as part of multiple-level surgery in patients with OSAS. Nasal surgery may help in OSA patients who do not tolerate CPAP therapy, when there is a local obstructive factor in the nose.

## Figures and Tables

**Table 1:** Nasal pathophysiology in the pathogenesis of OSAS.

Starling resistor model	Increased nasal resistance results in negative oropharyngeal pressure (suction force).
Instability of mouth breathing	Significant increase in nasal resistance generates higher fraction of oral breathing, leading to unstable airway.

Nasal ventilatory reflex	Decrease in nasal airflow results in less activation of nasal receptors and, consequently, inhibition of muscle tone, respiratory rate, and minute ventilation.

Nitric oxide (NO)	Decrease in nasal flow generates lower concentration of pulmonary NO with reduced ventilation-perfusion ratio.

**Table 2:** Studies on topical medication for the treatment of sleep disorders.

Study	Study design	Patients	Nasal pathology	Intervention	Results
Kiely et al., 2004 [40]	Double-blinded, controlled, randomized	10 snorers (mean AHI: 3), 13 OSAS (mean AHI: 26.5)	Allergic rhinitis without septal deviation	Fluticasone 100 mcg BD for four weeks versus placebo	Reduction in AHI and subjective nasal resistance. No difference in sleep architecture, snoring, or O_2_ saturation.

McLean et al., 2005 [44]	Cross-sectional, blinded	10 moderate to severe OSAS	Chronic nasal obstruction	Topical oxymetazoline (0.2 mg BD) and external nasal dilator versus placebo	Reduction in AHI, improved sleep architecture, and reduced oral breathing. No alterations in sleepiness.

Kerr et al., 1992 [43]	Cross-sectional, blinded	10 moderate to severe OSAS	Chronic nasal obstruction	Topical oxymetazoline and nasal dilator versus placebo	Mild improvement in arousal index. No alterations in AHI, O_2_ saturation, or sleepiness.

AHI: apnea-hypopnea index; OSAS: obstructive sleep apnea syndrome.

**Table 3:** Studies on nasal dilators for treatment of sleep disorders.

Study	Patients	Study design, intervention	Results	Commentaries
Bahammam et al., 1999 [46]	18 snorers, mean AHI: 8.9	Cross-sectional, Breathe Right versus placebo	Improvement on desaturation time and sleep architecture. No difference in AHI or arousal index.	Nasal dilation increased nasal cross-section area. No information regarding snoring.

Pevernagie et al., 2000 [47]	12 snorers, mean AHI: 6, chronic rhinitis and nasal obstruction	Cross-sectional, Breathe Right versus placebo	Reduction of snoring. No difference in AHI, sleep architecture, or arousal index.	Nasal dilation significantly decreased nasal resistance.

Djupesland et al., 2001 [48]	18 snorers, mean AHI: 9.3, nocturnal nasal obstruction	Cross-sectional, Breathe Right versus placebo	No difference in O_2_ saturation, snoring, or sleep architecture. Increase of AHI.	Nasal dilation increased cross-sectional area and nasal volume.

Schönhofer et al., 2003 [49]	38 OSAS, in use of CPAP, mean AHI: 17.1	Cross-sectional, Nozovent versus placebo	CPAP pressure reduction. No difference in AHI or O_2_ saturation.	Nasal dilation was not controlled by objective or subjective measures.

Hoijer et al., 1992 [50]	10 OSAS, mean AHI: 18	Cross-sectional, Nozovent versus placebo	Reduction of snoring and O_2_ saturation. No improvement on hypersomnolence.	Nasal dilation increased nasal airflow.

AHI: apnea-hypopnea index; OSAS: obstructive sleep apnea syndrome; CPAP: continuous positive airway pressure.

## References

[1] Hippocrates L. (1717). *De Morbus Popularibus*.

[2] Georgalas C. (2011). The role of the nose in snoring and obstructive sleep apnoea: an update. *European Archives of Oto-Rhino-Laryngology*.

[3] Fairbanks D. N. F. (1985). Effect of nasal surgery on snoring. *Southern Medical Journal*.

[4] Craig T. J., Teets S., Lehman E. B., Chinchilli V. M., Zwillich C. (1998). Nasal congestion secondary to allergic rhinitis as a cause of sleep disturbance and daytime fatigue and the response to topical nasal corticosteroids. *Journal of Allergy and Clinical Immunology*.

[5] Atkins M., Taskar V., Clayton N., Stone P., Woodcock A. (1994). Nasal resistance in obstructive sleep apnea. *Chest*.

[6] Lofaso F., Coste A., D'Ortho M. P., Zerah-Lancner F., Delclaux C., Goldenberg F., Harf A. (2000). Nasal obstruction as a risk factor for sleep apnoea syndrome. *European Respiratory Journal*.

[7] Ferris B. G., Mead J., Opie L. H. (1964). Partitioning of respiratory flow resistance in man. *Journal of Applied Physiology*.

[8] Kohler M., Thurnheer R., Bloch K. E. (2006). Side-selective, unobtrusive monitoring of nasal airflow and conductance. *Journal of Applied Physiology*.

[9] Heetderks D. R. (1927). Observations on the reactions of normal nasal mucosa membrane. *The American Journal of the Medical Sciences*.

[10] Olsen K. O., Kern E. B. (1990). Nasal influences on snoring and obstructive sleep apnea. *Mayo Clinic Proceedings*.

[11] Hudgel D. W., Robertson D. W. (1984). Nasal resistance during wakefulness and sleep in normal man. *Acta Oto-Laryngologica*.

[12] Cole P., Haight J. S. J. (1984). Posture and nasal patency. *American Review of Respiratory Disease*.

[13] Cole P., Haight J. S. J. (1984). Mechanisms of nasal obstruction during sleep. *Laryngoscope*.

[14] Verse T., Pirsig W. (2003). Impact of impaired nasal breathing on sleep-disordered breathing. *Sleep and Breathing*.

[15] Smith P. L., Wise R. A., Gold A. R., Schwartz A. R., Permutt S. (1988). Upper airway pressure-flow relationships in obstructive sleep apnea. *Journal of Applied Physiology*.

[16] Park S. S. (1993). Flow-regulatory function of upper airway in health and disease: a unified pathogenetic view of sleep-disordered breathing. *Lung*.

[17] Kuna S. T., Remmers J. E. (1985). Neural and anatomic factors related to upper airway occlusion during sleep. *Medical Clinics of North America*.

[18] Tanaka Y., Honda Y. (1989). Nasal obstruction as a cause of reduced PCO_2_ and disordered breathing during sleep. *Journal of Applied Physiology*.

[19] McNicholas W. T., Coffey M., Boyle T. (1993). Effects of nasal airflow on breathing during sleep in normal humans. *American Review of Respiratory Disease*.

[20] Douglas N. J., White D. P., Weil J. V., Zwillich C. W. (1983). Effect of breathing route on ventilation and ventilatory drive. *Respiration Physiology*.

[21] White D. P., Cadieux R. J., Lombard R. M., Bixler E. O., Kales A., Zwillich C. W. (1985). The effects of nasal anesthesia on breathing during sleep. *American Review of Respiratory Disease*.

[22] Berry R. B., Kouchi K. G., Bower J. L., Light R. W. (1995). Effect of upper airway anesthesia on obstructive sleep apnea. *American Journal of Respiratory and Critical Care Medicine*.

[23] Lundberg J. (1996). Airborne nitric oxide: Inflammatory marker and aerocrine messenger in man. *Acta Physiologica Scandinavica*.

[24] Blitzer M. L., Loh E., Roddy M. A., Stamler J. S., Creager M. A. (1996). Endothelium-derived nitric oxide regulates systemic and pulmonary vascular resistance during acute hypoxia in humans. *Journal of the American College of Cardiology*.

[25] Djupesland P. G., Chatkin J. M., Qian W., Cole P., Zamel N., McClean P., Furlott H., Haight J. S. J. (1999). Aerodynamic influences on nasal nitric oxide output measurements. *Acta Oto-Laryngologica*.

[26] Haight J. S. J., Djupesland P. G. (2003). Nitric oxide (NO) and obstructive sleep apnea (OSA). *Sleep and Breathing*.

[27] Suratt P. M., Turner B. L., Wilhoit S. C. (1986). Effect of intranasal obstruction on breathing during sleep. *Chest*.

[28] Lavie P., Fischel N., Zomer J., Eliaschar I. (1983). The effects of partial and complete mechanical occlusion of the nasal passages on sleep structure and breathing in sleep. *Acta Oto-Laryngologica*.

[29] Young T., Finn L., Kim H. (1997). Nasal obstruction as a risk factor for sleep-disordered breathing. The University of Wisconsin Sleep and Respiratory Research Group. *Journal of Allergy and Clinical Immunology*.

[30] Basner R. C., Simon P. M., Schwartzstein R. M., Weinberger S. E., Woodrow Weiss J. (1989). Breathing route influences upper airway muscle activity in awake normal adults. *Journal of Applied Physiology*.

[31] Shi Y.-X., Seto-Poon M., Wheatley J. R. (1998). Breathing route dependence of upper airway muscle activity during hyperpnea. *Journal of Applied Physiology*.

[32] Williams J. S., Janssen P. L., Fuller D. D., Fregosi R. F. (2000). Influence of posture and breathing route on neural drive to upper airway dilator muscles during exercise. *Journal of Applied Physiology*.

[33] Settipane R. A., Charnock D. R. (2007). Epidemiology of rhinitis: allergic and nonallergic. *Clinical Allergy and Immunology*.

[34] Ferguson B. J. (2004). Influences of allergic rhinitis on sleep. *Otolaryngology: Head and Neck Surgery*.

[35] McNicholas W. T., Tarlo S., Cole P., Zamel N., Rutherford R., Griffin D., Phillipson E. A. (1982). Obstructive apneas during sleep in patients with seasonal allergic rhinitis. *American Review of Respiratory Disease*.

[36] Stuck B. A., Czajkowski J., Hagner A.-E., Klimek L., Verse T., Hörmann K., Maurer J. T. (2004). Changes in daytime sleepiness, quality of life, and objective sleep patterns in seasonal allergic rhinitis: a controlled clinical trial. *Journal of Allergy and Clinical Immunology*.

[37] Kalpaklıoğlu, A. F., Kavut A. B., Ekici M. (2009). Allergic and nonallergic rhinitis: the threat for obstructive sleep apnea. *Annals of Allergy, Asthma & Immunology*.

[38] McNicholas W. T. (2008). The nose and OSA: variable nasal obstruction may be more important in pathophysiology than fixed obstruction. *European Respiratory Journal*.

[39] Kotecha B. (2011). The nose, snoring and obstructive sleep apnoea. *Rhinology*.

[40] Kiely J. L., Nolan P., McNicholas W. T. (2004). Intranasal corticosteroid therapy for obstructive sleep apnoea in patients with co-existing rhinitis. *Thorax*.

[41] Cassisi N. J., Biller H. F., Ogura J. H. (1971). Changes in arterial oxygen tension and pulmonary mechanics with the use of posterior packing in epistaxis: a preliminary report.. *Laryngoscope*.

[42] Craig T. J., Hanks C. D., Fisher L. H. (2005). How do topical nasal corticosteroids improve sleep and daytime somnolence in allergic rhinitis?. *Journal of Allergy and Clinical Immunology*.

[43] Kerr P., Millar T., Buckle P., Kryger M. (1992). The importance of nasal resistance in obstructive sleep apnea syndrome. *Journal of Otolaryngology*.

[44] McLean H. A., Urton A. M., Driver H. S., Tan A. K. W., Day A. G., Munt P. W., Fitzpatrick M. F. (2005). Effect of treating severe nasal obstruction on the severity of obstructive sleep apnoea. *European Respiratory Journal*.

[45] Clarenbach C. F., Kohler M., Senn O., Thurnheer R., Bloch K. E. (2008). Does nasal decongestion improve obstructive sleep apnea?. *Journal of Sleep Research*.

[46] Bahammam A. S., Tate R., Manfreda J., Kryger M. H. (1999). Upper airway resistance syndrome: effect of nasal dilation, sleep stage, and sleep position. *Sleep*.

[47] Pevernagie D., Hamans E., Van Cauwenberge P., Pauwels R. (2000). External nasal dilation reduces snoring chronic rhinitis patients: a randomized controlled trial. *European Respiratory Journal*.

[48] Djupesland P. G., Skatvedt O., Borgersen A. K. (2001). Dichotomous physiological effects of nocturnal external nasal dilation in heavy snorers: the answer to a rhinologic controversy?. *American Journal of Rhinology*.

[49] Schönhofer B., Kerl J., Suchi S., Köhler D., Franklin K. A. (2003). Effect of nasal valve dilation on effective CPAP level in obstructive sleep apnea. *Respiratory Medicine*.

[50] Hoijer U., Ejnell H., Hedner J., Petruson B., Eng L. B. (1992). The effects of nasal dilation on snoring and obstructive sleep apnea. *Archives of Otolaryngology—Head and Neck Surgery*.

[51] Kohler M., Bloch K. E., Stradling J. R. (2009). The role of the nose in the pathogenesis of obstructive sleep apnea. *Current Opinion in Otolaryngology & Head & Neck Surgery*.

[52] Li H. Y., Lee L. A., Wang P. C., Chen N. H., Lin Y., Fang T. J. (2008). Nasal surgery for snoring in patients with obstructive sleep apnea. *Laryngoscope*.

[53] Li H.-Y., Lin Y., Chen N.-H., Lee L.-A., Fang T.-J., Wang P.-C. (2008). Improvement in quality of life after nasal surgery alone for patients with obstructive sleep apnea and nasal obstruction. *Archives of Otolaryngology: Head and Neck Surgery*.

[54] Verse T., Maurer J. T., Pirsig W. (2002). Effect of nasal surgery on sleep-related breathing disorders. *Laryngoscope*.

[55] Morinaga M., Nakata S., Yasuma F., Noda A., Yagi H., Tagaya M., Sugiura M., Teranishi M., Nakashima T. (2009). Pharyngeal morphology: a determinant of successful nasal surgery for sleep apnea. *The Laryngoscope*.

[56] Koutsourelakis I., Georgoulopoulos G., Perraki E., Vagiakis E., Roussos C., Zakynthinos S. G. (2008). Randomised trial of nasal surgery for fixed nasal obstruction in obstructive sleep apnoea. *European Respiratory Journal*.

[57] McArdle N., Devereux G., Heidarnejad H., Engleman H. M., Mackay T. W., Douglas N. J. (1999). Long-term use of CPAP therapy for sleep apnea/hypopnea syndrome. *American Journal of Respiratory and Critical Care Medicine*.

[58] Hoffstein V., Viner S., Mateika S., Conway J. (1992). Treatment of obstructive sleep apnea with nasal continuous positive airway pressure: patient compliance, perception of benefits, and side effects. *The American Review of Respiratory Disease*.

[59] Tárrega J., Mayos M., Montserrat J. R., Fabra J. M., Morante F., Cáliz A., Sanchis J. (2003). Nasal resistance and continuous positive airway pressure treatment for sleep apnea/hypopnea syndrome. *Archivos de Bronconeumologia*.

[60] Haddad F. L. M., Vidigal T. D. A., Mello-Fujita L., Cintra F. D., Gregório L. C., Tufik S., Bittencourt L. (2013). The influence of nasal abnormalities in adherence to continuous positive airway pressure device therapy in obstructive sleep apnea patients. *Sleep and Breathing*.

[61] Morris L. G., Setlur J., Burschtin O. E., Steward D. L., Jacobs J. B., Lee K. C. (2006). Acoustic rhinometry predicts tolerance of nasal continuous positive airway pressure: a pilot study. *American Journal of Rhinology*.

[62] Sugiura T., Noda A., Nakata S., Yasuda Y., Soga T., Miyata S., Nakai S., Koike Y. (2006). Influence of nasal resistance on initial acceptance of continuous positive airway pressure in treatment for obstructive sleep apnea syndrome. *Respiration*.

[63] So Y. K., Dhong H.-J., Kim H. Y., Chung S.-K., Jang J.-Y. (2009). Initial adherence to autotitrating positive airway pressure therapy: Influence of upper airway narrowing. *Clinical and Experimental Otorhinolaryngology*.

[64] Powell N. B., Zonato A. I., Weaver E. M., Li K., Troell R., Riley R. W., Guilleminault C. (2001). Radiofrequency treatment of turbinate hypertrophy in subjects using continuous positive airway pressure: a randomized, double-blind, placebo-controlled clinical pilot trial. *Laryngoscope*.

[65] Friedman M., Tanyeri H., Lim J. W., Landsberg R., Vaidyanathan K., Caldarelli D. (2000). Effect of improved nasal breathing on obstructive sleep apnea. *Otolaryngology: Head and Neck Surgery*.

[66] Nakata S., Noda A., Yagi H., Yanagi E., Mimura T., Okada T., Misawa H., Nakashima T. (2005). Nasal resistance for determinant factor of nasal surgery in CPAP failure patients with obstructive sleep apnea syndrome. *Rhinology*.

